# The Role of GLP-1 Signaling in Hypoglycemia due to Hyperinsulinism

**DOI:** 10.3389/fendo.2022.863184

**Published:** 2022-03-24

**Authors:** Melinda Danowitz, Diva D. De Leon

**Affiliations:** ^1^Division of Endocrinology and Diabetes, Children’s Hospital of Philadelphia, Philadelphia, PA, United States; ^2^Department of Pediatrics, Perelman School of Medicine at the University of Pennsylvania, Philadelphia, PA, United States

**Keywords:** incretin, GLP-1, GIP, hyperinsulinism, dumping syndrome, bariatric surgery, fundoplication

## Abstract

Incretin hormones play an important role in the regulation of glucose homeostasis through their actions on the beta cells and other tissues. Glucagon-like peptide-1 (GLP-1) and glucose dependent insulinotropic polypeptide (GIP) are the two main incretins and are secreted by enteroendocrine L- and K-cells, respectively. New evidence suggests that incretin hormones, particularly GLP-1, play a role in the pathophysiology of hyperinsulinemic hypoglycemia. In individuals with acquired hyperinsulinemic hypoglycemia after gastrointestinal surgery, including Nissen fundoplication and gastric bypass surgery, the incretin response to a meal is markedly increased and antagonism of the GLP-1 receptor prevents the hyperinsulinemic response. In individuals with congenital hyperinsulinism due to inactivating mutations in the genes encoding the beta cell K_ATP_ channels, the GLP-1 receptor antagonist, exendin-(9-39), increases fasting plasma glucose and prevents protein-induced hypoglycemia. Studies in human and mouse islets lacking functional K_ATP_ channels have demonstrated that the effect on plasma glucose is at least in part mediated by inhibition of insulin secretion resulting from lower cytoplasmic cAMP levels. The understanding of the role of incretin hormones in the pathophysiology of hyperinsulinemic hypoglycemia is important for the exploration of the GLP-1 receptor as a therapeutic target for these conditions. In this article, we will review incretin physiology and evidence supporting a role of the incretin hormones in the pathophysiology of hyperinsulinemic hypoglycemia, as well as results from proof-of concept studies exploring a therapeutic approach targeting the GLP-1 receptor to treat hyperinsulinemic hypoglycemia.

## Introduction

Incretins are hormones secreted by intestinal cells in response to ingested nutrients that play a role in the regulation of glucose homeostasis. Glucagon-like-peptide-1 (GLP-1) and glucose-dependent insulinotropic polypeptide (GIP) are the two main incretins. Incretins effects on glucose homeostasis are mediated by several mechanisms, the most prominent being potentiation of insulin secretion. Effects of endogenous GLP-1 have been better characterized using the receptor antagonist exendin-(9-39). In this article, we will review incretin physiology and evidence supporting a role of GLP-1 in the pathophysiology of hyperinsulinemic hypoglycemia, as well as results from proof-of concept studies exploring a therapeutic approach targeting the GLP-1 receptor to treat hyperinsulinemic hypoglycemia.

## Incretin Physiology

GLP-1 is made and released by the enteroendocrine L-cells, which are found throughout the small and large intestines, with higher concentration in the ileum. GIP is secreted by the enteroendocrine K-cells concentrated in the proximal intestines, including the duodenum and jejunum (1, 2). Incretin secretion is stimulated by nutrient ingestion, the most potent activators being carbohydrate and lipid ingestion; protein is a weaker stimulus for incretin secretion (3). Levels of incretins increase rapidly after food administration *via* both neural and endocrine mediated factors (4). The secretion of GIP from K-cells is dependent on nutrient absorption, whereas L-cells can secrete GLP-1 triggered by glucose in the intestinal lumen. This observation is supported by studies in patients with disorders affecting gut absorption demonstrating a significant decrease in plasma GIP levels following a meal and normal to elevated GLP-1 levels (5). This phenomenon, however, can also be explained by increased glucose distribution to distal intestinal cells in patients with malabsorption, leading to higher luminal glucose concentrations in the distal intestines where GLP-1 secreting L-cells are highly concentrated (6). GLP-1 plasma levels rise within minutes of oral nutrient ingestion, suggesting that neural factors and taste receptors play a role in stimulating its secretion, in addition to direct contact with the enteroendocrine cells of the intestines (7). GIP and GLP-1 plasma levels are low in the fasting state, and increase post-prandially; GIP concentrations are higher than GLP-1 concentrations in both fasting and fed state (2). GIP plasma concentrations peak 30 minutes after a meal, and plasma GLP-1 rises within a few minutes and levels remain high for several hours after a meal. Both incretins are hydrolyzed by the enzyme dipeptidyl-peptidase 4 (DPP-IV).

**Figure 1:**
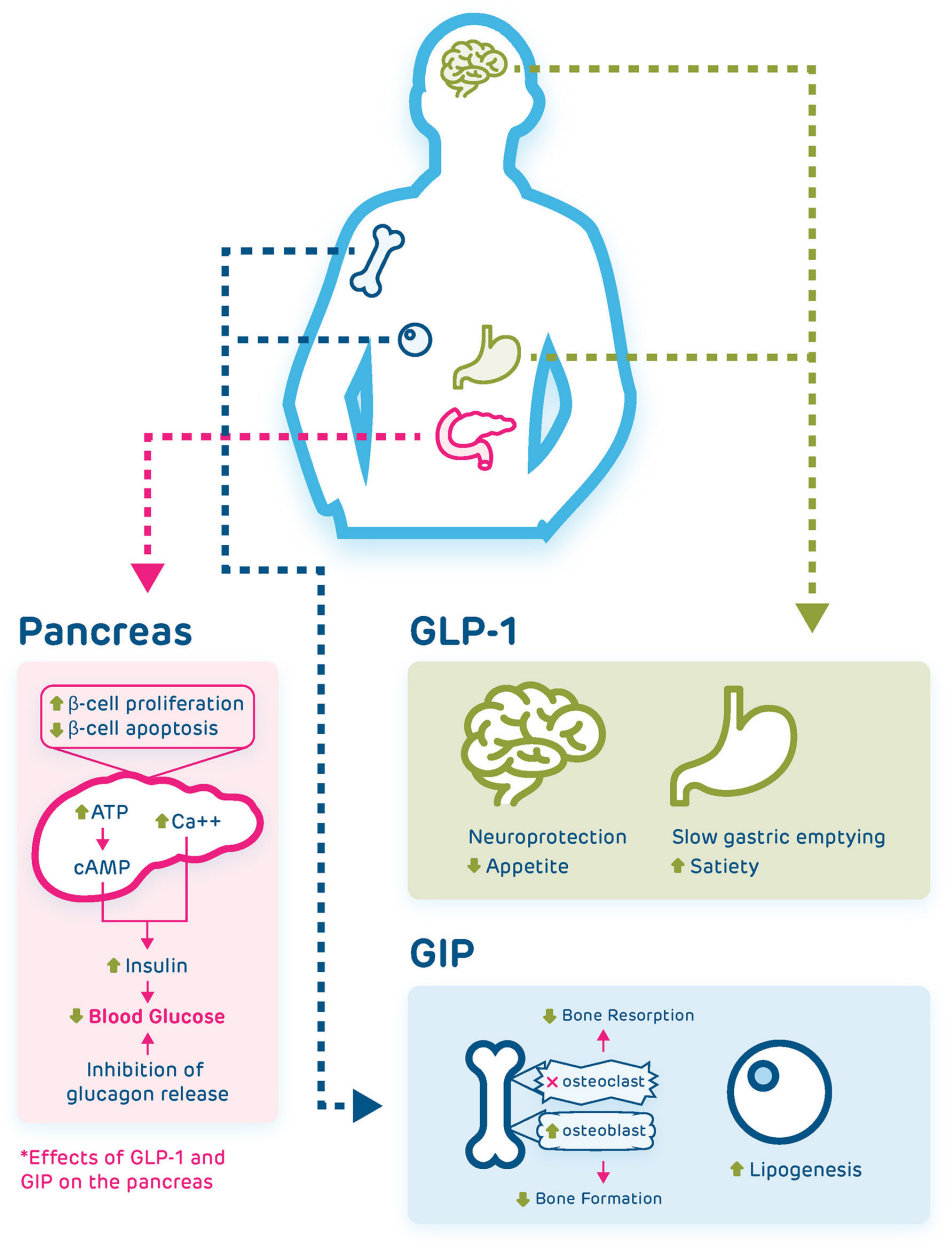
Pancreatic and extrapancreatic effects of GLP-1 and GIP. Both incretins act on the beta cells of the pancreas and lead to reduction of blood glucose *via* several mechanisms, including increasing intracellular calcium, induction of a cAMP mediated pathway, and inhibition of glucagon release. GLP-1 also acts on the brain where it has neuroprotective effects and suppresses appetite, and on the stomach where it leads to slowed gastric emptying and increased satiety. GIP acts on the bone where it inhibits osteoclast action and promotes osteoblast action, leading to increased bone formation, and acts on fat cells to induce lipogenesis.

Incretins act on G-protein coupled receptors on the beta cells of the pancreas, leading to a cAMP mediated pathway that ultimately results in insulin secretion (3). New research has also shown a separate pathway where low concentrations of GLP-1 lead to increased intracellular calcium and insulin secretion independent of the cAMP pathway (8) (**Figure 1**). In addition to its direct effects on beta cells, GIP also leads to increased glucagon secretion, and indirectly results in increased insulin secretion *via* communication between the alpha and beta cells of the pancreas (9). GLP-1 suppresses glucagon secretion *via* complex mechanisms, including stimulation of somatostatin secretion, which inhibits glucagon secretion, and indirectly *via* stimulation of insulin production, which then inhibits glucagon release (10).

Incretin action plays a large role on post-prandial secretion of insulin, and may account for up to 70% of hormone release (6). Thus, gut absorption of glucose leads to significantly greater rise in plasma insulin when compared to intravenous administration of glucose, due to incretin mediated insulin secretion (4), a phenomenon known as the “incretin effect”. Incretins also promote pancreatic beta cell proliferation and cell survival by decreasing apoptosis (6, 11). GLP-1 increases insulin secretion and synthesis as well as increases insulin sensitivity, induces beta cell growth, and inhibits glucagon secretion (1). In addition to post-prandial effects, GLP-1 suppresses glucagon in the fasting state, and can inhibit duodenal motility, leading to lower fasting plasma glucose (12). This suggests incretin effects that are independent of insulin action, as insulin levels remain low to undetectable in this state.

Both incretins have effects outside the pancreas. GLP-1 effects include slowing gastric emptying, increasing satiety, as well as cardioprotective effects (3, 13). In addition to secretion by intestinal cells, GLP-1 is produced in the central nervous system and can cross the blood brain barrier. Evidence suggests that GLP-1 has neuroprotective effects in addition to having effects on appetite regulation by decreasing appetite and increasing satiety (14, 15). GIP also has extra-pancreatic effects, including, stimulation of lipogenesis and stimulation of bone formation *via* inducing osteoblast activity and reduction of bone resorption *via* inhibition of osteoclasts (3) (**Figure 1**). The GLP-1 receptor antagonist, exendin-(9-39), has been used to demonstrate physiologic effects of endogenous GLP-1 in human studies. Exendin-(9-39) is a truncated form of exendin-4, a GLP-1 receptor agonist, isolated from the saliva of the gila monster. Exendin-(9-39) has been found to competitively bind to the human GLP-1 receptor (16). Some studies have shown that exendin-(9-39) also binds the GIP receptor (17, 18), however, Schirra and colleagues have demonstrated that doses of exendin-(9-39) that completely antagonize the insulinotropic effects of GLP-1 do not alter the insulinotropic activity of GIP *in vivo* in humans (16). Administration of exendin-(9-39) in healthy human subjects has been shown to decrease insulin secretion and increase glucose levels following meals, increase glucagon and glucose levels in the fasting state, and decrease insulin action with increased glucose following intravenous glucose administration (12, 19, 20), demonstrating *in vivo* that endogenous GLP-1 plays an important role on maintaining glucose homeostasis both in the fasting and in the postprandial state through its effects on pancreatic islets.

Because of their glucose lowering effects, therapies that enhance incretin actions through inhibiting degradation of endogenous incretins or through activation of the GLP-1 receptor are now in use for the treatment of type 2 diabetes (21, 22). In addition, pharmacologic doses of GLP-1 analogues have potent weight loss effects and are now in the market (23, 24). These pharmacologic studies have shown that these therapies are safe and well-tolerated and that they are not associated with overt hypoglycemia. However, GLP-1 administration has been shown to result in hypoglycemia when concomitantly administered with intravenous dextrose (25) or with sulfonylureas (26). What these two conditions may have in common is that the beta cells are in a depolarized state, thus, the glucose-dependency of GLP-1 insulinotropic effects is lost. Here we will discuss two pathologic conditions were GLP-1 has been associated with hypoglycemia, in congenital hyperinsulinism due to inactivating mutations in the K_ATP_ channels and in postprandial hypoglycemia after gastrointestinal surgery, where the delivery of nutrients to the small intestine is altered resulting in both hyperglycemia and an exaggerated secretion of GLP-1. In both conditions, one would hypothesized that the glucose-dependency of GLP-1 is also lost given the lack of functional K_ATP_ channels in the first, and the concomitant hyperglycemia in the second.

## Role of GLP-1 in Congenital Hyperinsulinism

Congenital hyperinsulinism is the most common cause of persistent hypoglycemia in neonates, infants, and children. Congenital hyperinsulinism is a heterogeneous condition in regards to genotype and phenotype. Over 12 different genetic loci have been associated with congenital hyperinsulinism but the most common genetic cause, which accounts for up to 60% of cases (27), are inactivating mutations in *ABCC8* and *KCNJ11*, which encode the two subunits of the beta cell K_ATP_ channel. Histologically, congenital hyperinsulinism can be diffuse, where all beta cells are affected, or focal, where there is a focal area of adenomatosis (28). Phenotypically, there are differences on triggers of hypoglycemia (fasting, protein consumption, exercise) and responsiveness to treatment, among the different types of hyperinsulinism (28).

Plasma incretin concentrations in both the fasting and fed state have been measured in patients with different forms of congenital hyperinsulinism. A study reported that there was no difference in post-prandial plasma incretin concentrations between diffuse and focal cases, and no difference in baseline plasma incretin concentration between focal, diffuse, and transient hyperinsulinism (29). Patients with atypical hyperinsulinism (defined as having no identified genetic mutation and with mosaic histomorphology) had significantly higher post-prandial plasma GLP-1 concentration when compared to other forms of hyperinsulinism (29). The study authors proposed that increased insulin levels in atypical hyperinsulinism may be driven by increased incretin action, specifically GLP-1, and propose that GLP-1 can be utilized as a biomarker to help identify and diagnose atypical hyperinsulinism (29), however, these findings need to be replicated in a larger population.

Studies in a mouse model of K_ATP_ hyperinsulinism, the most common and severe genetic subtype of hyperinsulinism, have shown that the GLP-1 receptor antagonist exendin-(9-39) significantly increases fasting plasma glucose and decreases the insulin to glucose ratio on the fasting state, effectively reversing the hyperinsulinemic hypoglycemia phenotype. These effects were mediated by reduction in islet cAMP concentration, as demonstrated by experiments *in vitro* in isolated *Sur1^-/-^* mouse islets (30). In addition to the effects of exendin-(9-39) on baseline islet cAMP and insulin secretion, exendin-(9-39) inhibited amino acid-stimulated insulin secretion, a key phenotypic feature of hyperinsulinism due to inactivating mutations in the K_ATP_ channels where consumption of protein leads to insulin secretion and hypoglycemia (30, 31).

The findings from mouse islets, have been replicated in human islets isolated from the pancreas of infants with diffuse K_ATP_ hyperinsulinism. Exendin-(9-39) effectively inhibited amino acid-stimulated insulin secretion from these islets. Furthermore, in adolescents and adults with K_ATP_ hyperinsulinism, a continuous intravenous infusion of exendin-(9-39) resulted in higher fasting plasma glucose concentrations and lower insulin to glucose ratio; plasma glucagon concentration was unaffected by exendin-(9-39) in this study. These studies suggest that GLP-1 and its receptor play a role in the pathophysiology of congenital hyperinsulinism and that targeting the GLP-1 receptor may be an effective treatment approach for this condition (32). In more recent studies, we have shown that exendin-(9-39) prevents both fasting and protein-induced hypoglycemia in children with K_ATP_ hyperinsulinism and decreases glucose infusion rate requirements in infants with K_ATP_ hyperinsulinism (33–35). Because of these promising results, exendin-(9-39) was granted breakthrough therapy designation for the treatment of congenital hyperinsulinism by the Food and Drug Administration, and studies to evaluate efficacy and safety of multiple dose regimens are under development (36).

## Role of GLP-1 in Hyperinsulinemic Hypoglycemia Secondary to Gastric Surgery

Plasma glucose homeostasis, particularly in the post-prandial state is altered after gastric surgery, including gastrectomy, fundoplication, and bariatric procedures (37). Severe postprandial hypoglycemia following an exaggerated insulin response to meals has been recognized as a consequence of these procedures. Incretins contribute to the post-prandial hypoglycemia *via* increased GLP-1 release potentiating insulin secretion.

### GLP-1 in Post-Prandial Hypoglycemia Following Fundoplication

Post-prandial hypoglycemia, also known as late dumping syndrome, is a relatively common phenomenon seen in children who undergo surgical procedures altering the anatomy or function of the gastrointestinal tract, particularly after Nissen Fundoplication or variants thereof. Approximately 25% of children who undergo Nissen Fundoplication experience post-prandial hypoglycemia (38). Nutrient ingestion, particularly if given through a gastrostomy, in children who underwent Nissen Fundoplication leads to rapid increase in plasma glucose as well as plasma insulin followed by hypoglycemia; this may be, in part, due to exaggerated GLP-1 secretion (39, 40). One study analyzed risk factors for developing dumping syndrome following fundoplication in children. Risk factors for dumping syndrome that were identified included fundoplication surgery within 1 year of age, presence of severe scoliosis, microgastria, and major cardiac abnormality (41).

Children with post-prandial hypoglycemia after Nissen fundoplication exhibit higher GLP-1 and insulin plasma concentrations, and lower plasma glucose nadir in response to an oral glucose tolerance test than controls. The authors proposed that the increased GLP-1 levels potentiated the insulin surge and contributed to the subsequent hypoglycemia. The children that participated in the study had appropriate suppression of insulin secretion in response to hypoglycemia and had a normal fasting tolerance, arguing against an underlying disorder of insulin secretion (39). Alterations on the delivery of nutrients to the small intestine resulting from the fundoplication, which decreases the accommodating capacity of the stomach, may explain the rapid rise in glucose and enhanced GLP-1 release.

In order to better characterize the role of endogenous GLP-1 in post-prandial hypoglycemia secondary to fundoplication, the insulin response to a standardized mixed meal in affected children was measured with concomitant administration of exendin-(9-39) or vehicle through a continuous intravenous infusion. This study found that the insulin surge following the meal was blunted with the administration of the GLP-1 antagonist, suggesting that GLP-1 plays an important role in the exaggerated insulin response seen in post-prandial hypoglycemia in children who had undergone a fundoplication (42). Glucagon concentration was higher during administration of exendin-(9-39) versus the vehicle condition in this study (42).

### GLP-1 in Post-Prandial Hypoglycemia Following Bariatric Surgery

A similar phenomenon of post-prandial hyperinsulinemic hypoglycemia is seen in adult patients after weight loss procedures, gastrectomy and Roux-en-Y gastric bypass, in particular; this is commonly referred to as dumping syndrome. Approximately 25% of patients who undergo total gastrectomy surgery experience subsequent dumping syndrome (43). Post-prandial hyperinsulinemic hypoglycemia in gastric bypass patients ameliorates with nutrient administration *via* the stomach (using a gastrostomy tube into the remnant stomach), rather than *via* the bypassed gastrointestinal tract. Post-prandial levels of insulin and GLP-1 normalized with nutrient administration into the stomach rather than the gastric bypass route, suggesting that altered nutrient delivery to the intestines contributes to dumping syndrome (44).

To better understand the timing of hormone secretion in relation to hypoglycemia in dumping syndrome, glucose, insulin, GLP-1, and norepinephrine levels were measured following a meal in patients with dumping syndrome, and compared to levels and timing of peak in control patients. Plasma insulin concentration peaked at 60 minutes in patients with dumping syndrome, and at 90 minutes in control patients. Plasma GLP-1 concentration peaked earlier and were higher in dumping syndrome patients; levels peaked at 20 minutes in patients with dumping syndrome and 30 minutes in control patients. There was a significant inverse correlation between plasma GLP-1 concentration shortly after a meal and plasma glucose concentration 2 hours post-prandially, coinciding with the plasma glucose nadir. Plasma glucagon was also measured in this study; baseline glucagon concentrations were similar between controls and subjects with dumping syndrome, and the glucagon levels peaked and remained elevated after 20 minutes in subjects with dumping syndrome. The study authors concluded that reactive hypoglycemia in dumping syndrome following bariatric surgery is caused by increased GLP-1 concentration potentiating insulin release (45).

Another similar study measured plasma incretin, insulin, and glucose concentration in gastrectomy patients compared to controls. In response to an oral glucose load, plasma GLP-1 concentration was increased in post-gastrectomy patients, when compared to controls with unaltered intestinal anatomy. There was a statistically significant correlation between GLP-1 increase and the presence of dumping syndrome. Plasma GLP-1 concentration peaked at 15 minutes post glucose load, and insulin peaked at 30 minutes, suggesting GLP-1 potentiated or triggered insulin secretion. The study found an inverse relation between the emptying time from the gastric pouch, and plasma GLP-1 and insulin concentration with the meal, indicating that quicker transit time led to increases in insulin and GLP-1 plasma concentration and subsequent post-prandial hypoglycemia (43).

Exendin-(9-39) given as an intravenous infusion to adult individuals with post-prandial hypoglycemia after gastric bypass surgery led to a blunted insulin response, suggesting that GLP-1 is responsible for the exaggerated insulin secretion in dumping syndrome (46). Exendin-(9-39) has shown to be effective in preventing hyperinsulinemic hypoglycemia in bariatric surgery patients, and may have therapeutic implications in this patient population (47, 48). One study assessed the effect of exendin-(9-39) administered as a subcutaneous injection in treating hypoglycemia following bariatric surgery by measuring glucose nadir and insulin peak during a mixed meal tolerance test, and by assessing glycemic control using home continuous glucose monitoring. Incidence of hypoglycemia measured by continuous glucose monitor and number of hypoglycemic events were decreased in the subjects treated with exendin-(9-39). There was a statistically significant increase in glucose nadir and decrease in insulin peak during the mixed meal tolerance test (49). Another study found a reduction in symptomatic hypoglycemic events, as well as decreased insulin peak and increased glucose nadir during oral glucose tolerance tests in subjects receiving exendin-(9-39) (50).

## Conclusions

The importance of incretin hormones on the regulation of glucose homeostasis through pancreatic and extra-pancreatic actions is well established. Because of their glucose lowering effects, therapies that enhance incretin actions through inhibiting degradation of endogenous incretins or through activation of the GLP-1 receptor are now in use for the treatment of type 2 diabetes. The recognition of a role for GLP-1 in the pathophysiology of congenital and acquired hyperinsulinemic hypoglycemia has brought forward the possibility of targeting the GLP-1 receptor for the treatment of this condition and promising progress has been made to this end.

## Author Contributions

MD performed literature review and wrote the manuscript. DDDL reviewed literature and edited the manuscript. All authors contributed to the article and approved the submitted version.

## Funding

MD receives fellowship funding from training grant 5T32DK063688-18.

## Conflict of Interest

DDDL is named as an inventor in patent USA Patent Number 9,616,108, 2017; USA Patent Number 9,821,031, 2017; Europe Patent Number EP 2120994, 2018; and Europe Patent Number EP2818181, 2019; which cover the use of exendin-(9-39) for treating hyperinsulinism and postprandial hypoglycemia. DL has donated all financial proceeds from these patents to the Children’s Hospital of Philadelphia. DL has served as a consultant for Zealand Pharma A/S, Crinetics Pharmaceuticals, Hanmi Pharmaceutical, Heptares Therapeutics, Poxel Pharma, Ultragenyx, and Eiger Pharma. She has received research funding unrelated to this project from Tiburio Therapeutics, Crinetics Pharmaceuticals, and Twist Pharma. DL owns stock options from Merck & Co.

The remaining author declares that the research was conducted in the absence of any commercial or financial relationships that could be construed as a potential conflict of interest.

## Publisher’s Note

All claims expressed in this article are solely those of the authors and do not necessarily represent those of their affiliated organizations, or those of the publisher, the editors and the reviewers. Any product that may be evaluated in this article, or claim that may be made by its manufacturer, is not guaranteed or endorsed by the publisher.
